# Construction and validation of a machine learning-based model for predicting pneumonia risk in patients with hemorrhagic stroke

**DOI:** 10.3389/fmed.2026.1869603

**Published:** 2026-07-24

**Authors:** Darong Lu, Wanting Shi, Li Wu, Luo Yefangxin, Yanjing Li, Qiong Qin, Runqin Huang, Yan Xiong, Xuemei Chen, Yunting Li, Wei Chen

**Affiliations:** 1 Department of Neurosurgery, Affiliated Hospital of Zunyi Medical University, Zunyi, Guizhou, China; 2 Department of Nursing, Affiliated Hospital of Zunyi Medical University, Zunyi, Guizhou, China; 3 School of Nursing, Zunyi Medical University, Zunyi, Guizhou, China

**Keywords:** early warning model, hemorrhagic stroke, interpretability, machine learning, pneumonia

## Abstract

**Objective:**

This study aimed to develop and validate a distinct, stable, and interpretable predictive model using machine learning techniques to identify individuals at high risk of pneumonia early after admission. The goal was to provide a potential quantitative reference for implementing preventive interventions in clinical practice.

**Methods:**

A retrospective nested case–control design was adopted. A total of 822 patients with hemorrhagic stroke admitted between January 2019 and October 2024 were enrolled. Feature selection was performed using LASSO regression to eliminate multicollinearity and identify key predictors. Five machine learning algorithms—logistic regression (LRC), gradient boosting classifier (GBC), random forest classifier (RFC), multilayer perceptron classifier (MLPC), and support vector machine classifier (SVC)—were employed to construct predictive models. Hyperparameters were optimized through 10-fold cross-validation and grid search. Model performance was comprehensively evaluated on an independent test set using metrics including area under the curve (AUC), accuracy, sensitivity, precision, and F1-score. Finally, SHAP (SHapley Additive exPlanations) values were applied to interpret the optimal model and elucidate the contribution of each feature to the prediction.

**Results:**

LASSO regression selected 14 key predictors from 57 initial variables. Among the five models, the logistic regression model achieved the best performance on the test set. SHAP-based interpretability analysis revealed that the most influential factors for pneumonia risk prediction were, in descending order: left lower limb muscle strength, total cholesterol (TC), right lower limb muscle strength, low-density lipoprotein cholesterol (LDL-C), white blood cell count (WBC), consciousness status, D-dimer, age, systolic blood pressure (SBP), and bleeding location.

**Conclusion:**

This study successfully developed a logistic regression-based predictive model for pneumonia risk in patients with hemorrhagic stroke. The model demonstrated favorable discrimination and stability. It provides an objective, quantitative basis for early identification of high-risk patients, stratified management, and precise prevention and control, supporting a shift from reactive to proactive complication management.

## 1 Introduction

Stroke has become one of the leading global causes of disability and death. Its high incidence, disability, and mortality rates impose a heavy medical and economic burden on families and society (1, 2). Hemorrhagic stroke (HS), recognized as the most devastating subtype among all stroke classifications, comprises approximately 15–20% of incident cases while contributing to over 45% of stroke-associated mortality (3). Beyond primary neurological injury, acute-phase complications represent pivotal factors that impede patient recovery and drive mortality rates. Among these, hospital-acquired pneumonia (HAP) stands out as one of the most prevalent and impactful comorbidities. According to the latest data from the China National Stroke Surveillance (CNSS) (4), the incidence of HAP in patients with hemorrhagic stroke reaches 34.6%, a rate significantly higher than that observed in ischemic stroke. The onset of pneumonia not only prolongs hospital stays and increases direct medical costs, but also exponentially elevates the risk of short-term mortality (5, 6). Owing to a convergence of critical pathophysiological changes—namely impaired consciousness, compromised swallowing reflexes, deficient cough and sputum clearance, and prolonged immobilization—patients with hemorrhagic stroke are exceptionally vulnerable to developing hospital-acquired pneumonia (7). Notably, pneumonia in this population often manifests with insidious onset and atypical clinical presentations (8). Conventional clinical scoring systems (e.g., AIS-APS, ICH scores) primarily emphasize neurological deficits while lacking sensitivity in predicting infection risk, thereby failing to meet the need for early and accurate warning (9, 10). Consequently, there is an unmet clinical imperative for an objective tool capable of rapidly identifying high-risk individuals upon admission using routine data, facilitating a paradigm shift from reactive antimicrobial therapy to proactive preventive intervention. In recent years, machine learning (ML) has demonstrated considerable promise in disease risk prediction, prognostic evaluation, and clinical decision support by virtue of its capacity to mine high-dimensional clinical data, decode non-linear associations, and perform adaptive modelin (11, 12). ML algorithms have been shown to outperform traditional regression models in forecasting venous thromboembolism (VTE) and sepsis (13). However, studies focusing specifically on hemorrhagic stroke-associated pneumonia (HSAP) remain scarce and exhibit notable limitations (14, 15): inadequate feature selection protocols, where insufficient variables or multicollinearity compromise model generalizability; prominent “black box” opacity, lacking interpretability analyses essential for clinician trust; and limited clinical translatability, often ignoring dynamic therapeutic impacts (e.g., use of dehydrating agents or sedatives) on infection risk. To address these gaps, the present study enrolled patients with hemorrhagic stroke, systematically curating baseline characteristics, laboratory findings, and treatment-related indicators. We utilized LASSO regression for feature selection, developed and compared multiple ML models, and employed SHAP values to interpret the optimal model. This study aims to construct a precise, stable, and clinically applicable prediction tool to facilitate early identification of high-risk patients, guide individualized prophylaxis, and ultimately improve overall outcomes.

## 2 Materials and methods

### 2.1 Study subjects

This study adopted a retrospective nested case-control design. We first constructed a source cohort comprising 2,896 patients with hemorrhagic stroke who met the inclusion and exclusion criteria between January 2019 and October 2024. Of these, 454 were diagnosed with pneumonia during hospitalization. To enhance statistical power, risk set sampling was employed: all 454 pneumonia cases were assigned to the case group, and 368 controls were systematically sampled from non-pneumonia patients admitted during the same period, matched for admission year and age strata. This yielded a final analytic dataset of 822 patients. Due to the nested design, the proportion of pneumonia in the analytic sample was significantly higher than the true incidence in the source population. Consequently, model outputs should be interpreted as relative risk rankings for stratifying high- versus low-risk populations, rather than as precise absolute probabilities. To ensure real-world generalizability, a single-center temporal validation strategy was implemented, with October 1, 2023, as the cutoff. The training (*n* = 492, 60%) and validation (*n* = 165, 20%) sets consisted exclusively of historical cases prior to this date, while the test set (*n* = 165, 20%) comprised only patients admitted from October 1, 2023, to October 31, 2024. Stratified random sampling (stratified by pneumonia status) ensured consistent distribution across subsets. Crucially, the test set represented strictly future data, held entirely isolated during model training and hyperparameter tuning, and used solely for final blinded evaluation. This approach effectively simulated real-world clinical performance, mitigating data leakage and optimistic bias.

### 2.2 Inclusion and exclusion criteria

#### 2.2.1 Inclusion and exclusion criteria

Inclusion criteria: ➀Patients diagnosed with hemorrhagic stroke (including intracerebral hemorrhage and subarachnoid hemorrhage) by head CT or MRI; ➁Age ≥ 18 years; ➂Complete hospital medical records containing all baseline characteristics, treatment measures, and laboratory test results required for this study; ➃Patients and their families were informed about the study and provided signed informed consent.

Exclusion criteria: ➀Hospital stay < 24 h; ➁Presence of confirmed pneumonia at admission or pneumonia occurring within 48 h of admission; ➂Comorbid with other severe end-stage diseases (e.g., advanced cancer, severe liver/kidney failure) with short expected survival; ➃Patients combined with ischemic stroke; ➄Medical records with severe missing data, lacking key variables (e.g., outcome indicators or main predictive variables).

#### 2.2.2 Diagnostic criteria for hospital-acquired pneumonia

The primary outcome was defined as Hospital-Acquired Pneumonia (HAP), diagnosed strictly according to the criteria established by the U.S. Centers for Disease Control and Prevention (CDC) and the Chinese Guidelines for the Diagnosis and Treatment of Hospital-Acquired Pneumonia and Ventilator-Associated Pneumonia in Adults (2018 Edition) (16). All suspected cases were required to meet all of the following criteria:

➀ Clinical Manifestations: New-onset or progressive cough, sputum production, dyspnea, or wheezing; accompanied by at least one of the following: fever (> 38°C) or hypothermia (< 36°C); signs of lung consolidation or moist rales upon auscultation; peripheral white blood cell count > 10 × 10^9^/L or < 4 × 10^9^/L.
➁ Radiographic Evidence: Chest X-ray or computed tomography (CT) demonstrating new or progressive infiltrates, consolidation, or ground-glass opacities, after excluding other pulmonary conditions such as pulmonary edema, atelectasis, or pulmonary embolism.
➂ Timing: The diagnosis of pneumonia must have been established ≥ 48 h after hospital admission. Cases occurring within the first 48 h were classified as community-acquired or pre-existing infections and were excluded from the outcome analysis.
➃ Inclusion Scope: Both ventilator-associated pneumonia (VAP) and aspiration pneumonia were included in the definition of HAP for this study.

### 2.3 Research methods

#### 2.3.1 Data collection

Data were retrospectively collected by two master’s students in nursing who received standardized training, using the hospital’s electronic medical record system. The clinical data of patients meeting the inclusion and exclusion criteria were extracted, encompassing 57 risk factors across six domains: demographic characteristics, disease and treatment features, personal and past medical history, clinical signs and complications, therapeutic interventions, and laboratory parameters. For all clinical signs and laboratory indicators, only the first measurements recorded upon admission were used for analysis. To ensure clinical applicability and avoid temporal bias, all predictors were strictly defined as data available within the early admission period (within 48 h of hospitalization), including early interventions and the confirmed admission status. Strict adherence to privacy protection principles was maintained throughout the data collection process.

#### 2.3.2 Statistical analysis

Data were organized and entered using Excel. All statistical analyses were performed using SPSS 29.0 and the Python programming language. Continuous variables conforming to a normal distribution are presented as mean ± standard deviation, with intergroup comparisons made using the *t*-test; those not conforming to a normal distribution are presented as median (interquartile range) [M (P25, P75)], with intergroup comparisons made using the Mann-Whitney U test. Categorical variables are presented as number (percentage) [n (%)], with intergroup comparisons made using the chi-square test or Fisher’s exact test. The AUC, accuracy, sensitivity, precision, and F1 score were calculated to evaluate model performance. A *P* < 0.05 was considered statistically significant.

#### 2.3.3 Prediction model construction

A total of 57 potential risk factors were incorporated as predictors, and all modeling procedures were implemented using Python. To ensure methodological rigor and prevent data leakage, a “partition-first” principle was strictly enforced, wherein all data preprocessing and feature engineering steps were confined exclusively to the training set.

➀ Dataset partitioning and reproducibility: To ensure reproducibility, a fixed random seed (Random Seed = 42) was set. The entire cohort was pre-divided into a training set (*n* = 492, 60%), a validation set (*n* = 165, 20%), and a strictly held-out test set (*n* = 165, 20%). Notably, the test set served as an independent external dataset, remaining completely isolated throughout the entire model training and hyperparameter tuning process, and was reserved solely for final blinded evaluation.
➁ Missing data imputation: For variables with a missing rate of less than 20%, multiple imputation via chained equations (MICE) was performed. Crucially, the imputation model was fitted only using the distribution of the training set and then applied to the validation and test sets to prevent information leakage. Similarly, feature scaling or normalization was performed using parameters derived solely from the training set.
➂ Feature selection: To mitigate multicollinearity, Least Absolute Shrinkage and Selection Operator Least Absolute Shrinkage and Selection Operator (LASSO) regression was applied to the 57 variables within the training set (*n* = 492). The optimal penalty coefficient (λ) was determined via 10-fold cross-validation, retaining only variables with non-zero coefficients for subsequent modeling.
➃ Model construction and tuning: Using the LASSO-selected features, five machine learning classifiers were constructed: Logistic Regression (LRC), Gradient Boosting Classifier (GBC), Random Forest Classifier (RFC), Multi-Layer Perceptron (MLPC), and Support Vector Classifier (SVC). During training, 10-fold cross-validation combined with class weight adjustment and Randomized Search was utilized to optimize hyperparameters, thereby enhancing model accuracy and stability.
➄ Model evaluation and interpretation: The performance of all five models was evaluated on the fully isolated test set. Core metrics included the Area Under the Receiver Operating Characteristic Curve (AUC-ROC), accuracy, sensitivity, precision, and the F1-score. The optimal model was selected based on comprehensive metric comparison. Finally, SHAP (SHapley Additive exPlanations) values were employed to interpret the optimal model and quantify the contribution of each feature to the predictions.

### 2.4 Data quality control

All clinical data were retrospectively collected from the hospital’s electronic medical record system by two uniformly trained nursing master’s students to ensure standardized data extraction. After collection, a third researcher performed cross-checking and logical error screening. Data with doubts or anomalies were re-checked against the original medical records. Throughout the data collection and analysis process, all patient personal information was anonymized, using only de-identified data to fully protect patient privacy. This study explicitly states that all data were used solely for this research.

### 2.5 Ethics and privacy

This study strictly adhered to medical research ethics guidelines and was approved by the Biomedical Ethics Committee of the Affiliated Hospital of Zunyi Medical University (Approval No.: KLLY-2023-122).

## 3 Results

### 3.1 Baseline characteristics and comparative analysis

The final analysis dataset included 822 HS patients, comprising 454 pneumonia patients and 368 non-pneumonia patients. Comparison of baseline characteristics between the two groups (Table 1) showed that the pneumonia and non-pneumonia groups had statistically significant differences (*P* < 0.05) in age, surgical approach, hypertension, coronary heart disease, liver and kidney disease, gastric mucosal lesions/gastrointestinal bleeding, deep vein thrombosis (DVT), respiratory failure, hyponatremia, admission VTE score, left lower limb muscle strength, right lower limb muscle strength, consciousness disorder status, head drainage tube, central venous catheterization, invasive arterial blood pressure monitoring, artificial airway, enteral nutrition, blood transfusion, persistent fever, use of diuretics, use of sedative drugs, D-dimer, white blood cell count, hemoglobin, hematocrit, C-reactive protein, prothrombin time, thrombin time, fibrinogen, blood glucose, and low-density lipoprotein cholesterol.

**TABLE 1:** Comparison of Baseline Characteristics of Included Patients [n(%), M(P25, P75)].

Variables	Pneumonia (*n* = 454)	Non-pneumonia (*n* = 368)	*t/χ* ^2^ */Z*	*P*
Gender			1.651	0.199
Male	270 (59.47)	235 (63.86)		
Female	184 (40.53)	133 (36.14)		
Age (years)	59 (52, 68)	57 (49, 68)	−2.902	0.004
Height	163 (159, 166)	163 (160, 166)	−0.239	0.811
Weight	64 (57, 70)	63 (57, 70)	−0.213	0.831
Body mass index (BMI)	23.78 (22.01, 25.68)	23.63 (22.02, 25.54)	−0.357	0.721
Head drainage tube	116 (25.55)	40 (10.87)	28.490	< 0.001
Central venous catheterization	128 (28.19)	40 (10.87)	37.514	< 0.001
Invasive arterial blood pressure monitoring	166 (36.56)	78 (21.20)	22.999	< 0.001
Enteral nutrition	192 (42.29)	72 (19.57)	48.147	< 0.001
Blood transfusion	42 (9.25)	8 (2.17)	17.820	< 0.001
Persistent fever	74 (16.30)	15 (4.08)	31.454	< 0.001
Surgical approach			43.977	< 0.001
No surgery	192 (42.29)	196 (53.26)		
Interventional	133 (29.30)	136 (36.96)		
Non-interventional	129 (28.41)	36 (9.78)		
Bleeding location			3.418	0.636
Basal ganglia	210 (46.26)	154 (41.85)		
Cerebral lobar	47 (10.35)	52 (14.13)		
Brainstem	26 (5.73)	20 (5.43)		
Ventricle	9 (1.98)	8 (2.17)		
Cerebellum	19 (4.19)	15 (4.08)		
Subarachnoid space	143 (31.50)	119 (32.34)		
Hypertension			11.881	0.008
No	109 (24.01)	128 (34.78)		
Grade I	37 (8.15)	22 (5.98)		
Grade II	62 (13.66)	43 (11.68)		
Grade III	246 (54.19)	175 (47.55)		
Coronary heart disease	29 (6.39)	11 (2.99)	5.071	0.024
Respiratory failure	107 (23.57)	12 (3.26)	67.699	< 0.001
Consciousness status			107.757	< 0.001
Conscious	168 (37.00)	258 (70.11)		
Somnolence	138 (30.40)	70 (19.02)		
Confusion	9 (1.98)	11 (2.99)		
Lethargy	22 (4.85)	9 (2.45)		
Coma	117 (25.77)	20 (5.43)		
Admission VTE score	6 (6, 7)	6 (6, 7)	−2.224	0.026
Left lower limb muscle strength (grade)	4 (2, 5)	5 (4, 5)	−9.424	< 0.001
Right lower limb muscle strength (grade)	4 (2, 5)	5 (4, 5)	−9.476	< 0.001
Thrombin time (s)	17 (16.1, 17.5)	17.2 (16.7, 17.5)	−3.733	< 0.001
Hyponatremia	28 (6.17)	45 (12.23)	9.227	0.002
Liver and kidney disease	81 (17.84)	20 (5.43)	29.029	< 0.001
Gastric mucosal lesions/Gastrointestinal bleeding	87 (19.16)	15 (4.08)	42.564	< 0.001
DVT	131 (28.85)	21 (5.71)	72.258	< 0.001
Artificial airway	188 (41.41)	58 (15.76)	63.761	< 0.001
Use of diuretics	140 (30.84)	197 (53.53)	43.280	< 0.001
Use of sedative drugs	146 (32.16)	35 (9.51)	60.714	< 0.001
Hemoglobin (g/L)	122 (112, 135)	131 (119, 139)	−4.917	< 0.001
Prothrombin time (s)	10.5 (10.1, 11.2)	10.4 (10.1, 10.8)	−2.632	0.008
Fibrinogen (g/L)	3.13 (2.75, 4.44)	2.91 (2.65, 3.48)	−4.469	< 0.001
Hematocrit (%)	0.38(0.34, 0.41)	0.4 (0.36, 0.42)	−4.118	< 0.001
D-dimer (mg/L)	1.18 (0.73, 2.34)	0.82 (0.53, 1.24)	−7.719	< 0.001
White blood cell count ( × 10^9^/L)	10.01 (8.24, 12.57)	8.64 (7.06, 10.38)	−6.953	< 0.001
C-reactive protein (mg/L)	9.68 (6.68, 37.41)	8.37 (3.58, 9.96)	−6.781	< 0.001
Thrombin time (s)	17 (16.1, 17.5)	17.2 (16.7, 17.5)	−3.733	< 0.001
Low-density lipoprotein cholesterol (mmol/L)	2.6 (2.26, 2.8)	2.68 (2.41, 2.86)	−3.658	< 0.001

“−” indicates Fisher’s exact probability method was used. Continuous variables are presented as mean ± SD or median (IQR); categorical variables are presented as n (%). *P* < 0.05 indicates statistical significance. DVT: Deep Vein Thrombosis. All treatment and intervention variables listed were used exclusively for baseline description and were excluded from the predictive modeling to avoid data leakage. The variables listed above constitute the core set for analysis. Additional variables are detailed in Supplementary material.

### 3.2 LASSO regression for predictive variable screening

**FIGURE 1:**
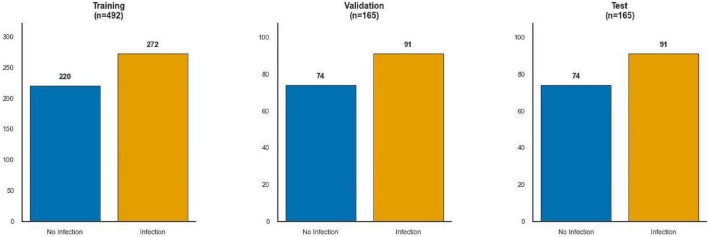
Outcome distribution in the training, validation, and test sets.

The dataset was randomly partitioned into training, validation, and test sets at a ratio of 60%:20%:20%. As illustrated in Figure 1, the class distribution (pneumonia vs. non-pneumonia) was well preserved across all subsets. Statistical comparison confirmed no significant differences in the incidence of pneumonia among the three groups (*P* > 0.05), effectively ruling out model performance overestimation due to sampling bias.

**FIGURE 2:**
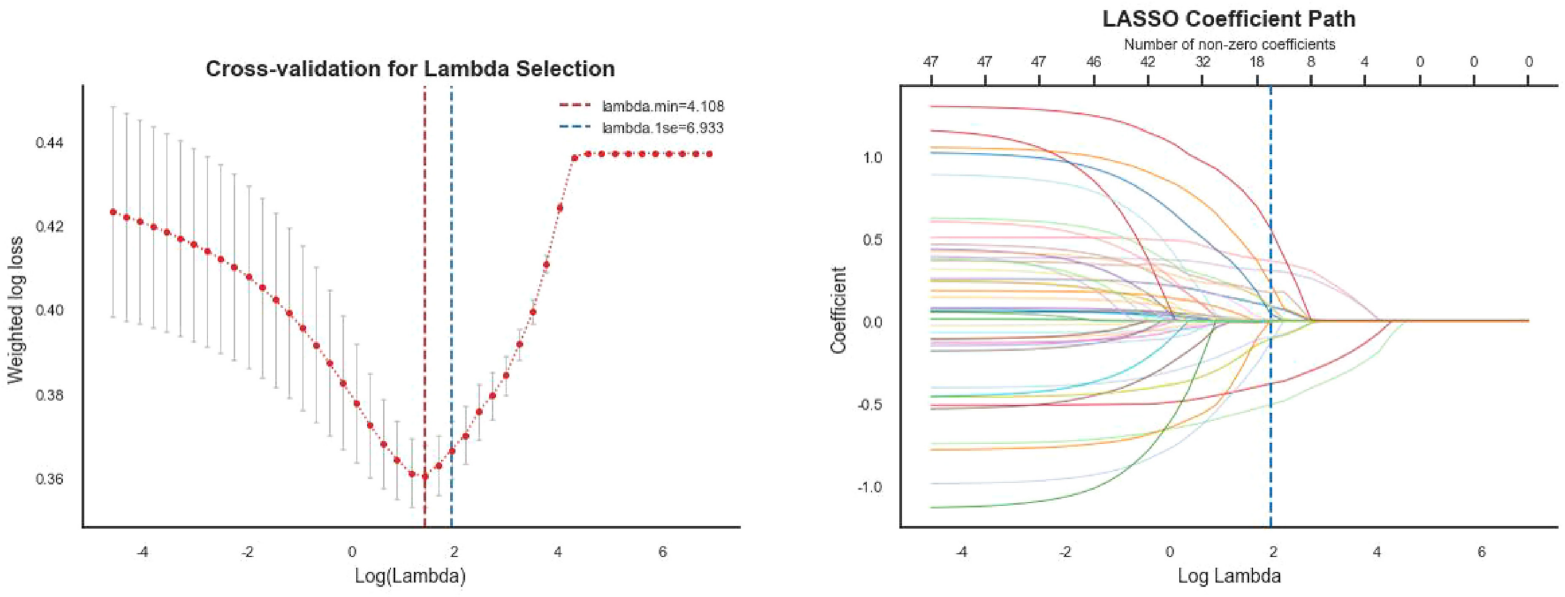
Feature selection using LASSO regression.

**FIGURE 3:**
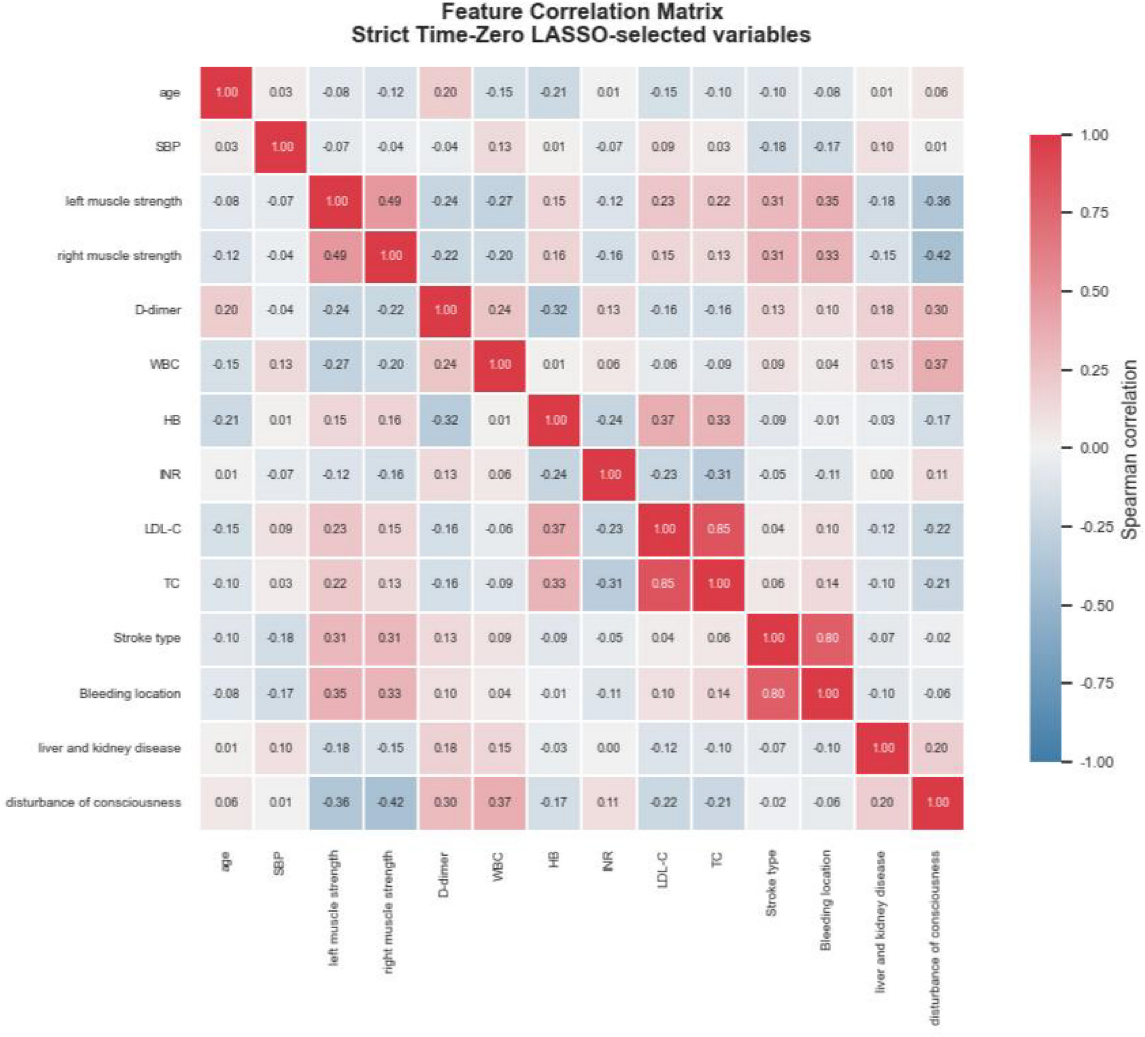
Correlation heatmap of the selected features.

To mitigate the impact of multicollinearity among the initial 57 variables on model performance, LASSO regression was employed for feature dimensionality reduction. To prevent prognostic information leakage, only baseline variables available at admission were included; all treatment, operative, and interventional variables occurring after admission were excluded. Feature selection was conducted exclusively within the training set using 10-fold cross-validation, applying the lambda.1se criterion to determine the optimal penalty coefficient (λ). As shown in Figure 2, as the regularization parameter λ increased, the coefficients of the independent variables were progressively compressed. Based on the 10-fold cross-validation results, lambda.1se was selected as the optimal penalty coefficient, balancing model parsimony and overfitting prevention. Ultimately, 14 key predictor variables with non-zero coefficients were identified (Figure 3), including: age, systolic blood pressure (SBP), left and right lower limb muscle strength, D-dimer, white blood cell count (WBC), hemoglobin (HB), international normalized ratio (INR), low-density lipoprotein cholesterol (LDL-C), total cholesterol (TC), stroke type, bleeding location, history of liver/kidney disease, and consciousness status. Figure 3 presents the Pearson correlation analysis among the 14 selected predictors. The results indicate that the absolute values of correlation coefficients among all variables were below 0.4, suggesting no severe multicollinearity issues. Notably, left and right lower limb muscle strength exhibited a strong positive correlation (*r* = 0.89, *P* < 0.001), aligning with the pathophysiological characteristics of neurological injury, where central lesions typically result in synchronous involvement of bilateral limbs. Additionally, D-dimer showed a moderate positive correlation with WBC (*r* = 0.41, *P* < 0.001), indicating that inflammatory states and coagulation dysfunction frequently coexist. This heatmap validates the effectiveness of LASSO regression in eliminating redundant information and retaining independent predictors, thereby establishing a robust foundation for subsequent model construction.

### 3.3 Machine learning model construction and evaluation

**FIGURE 4:**
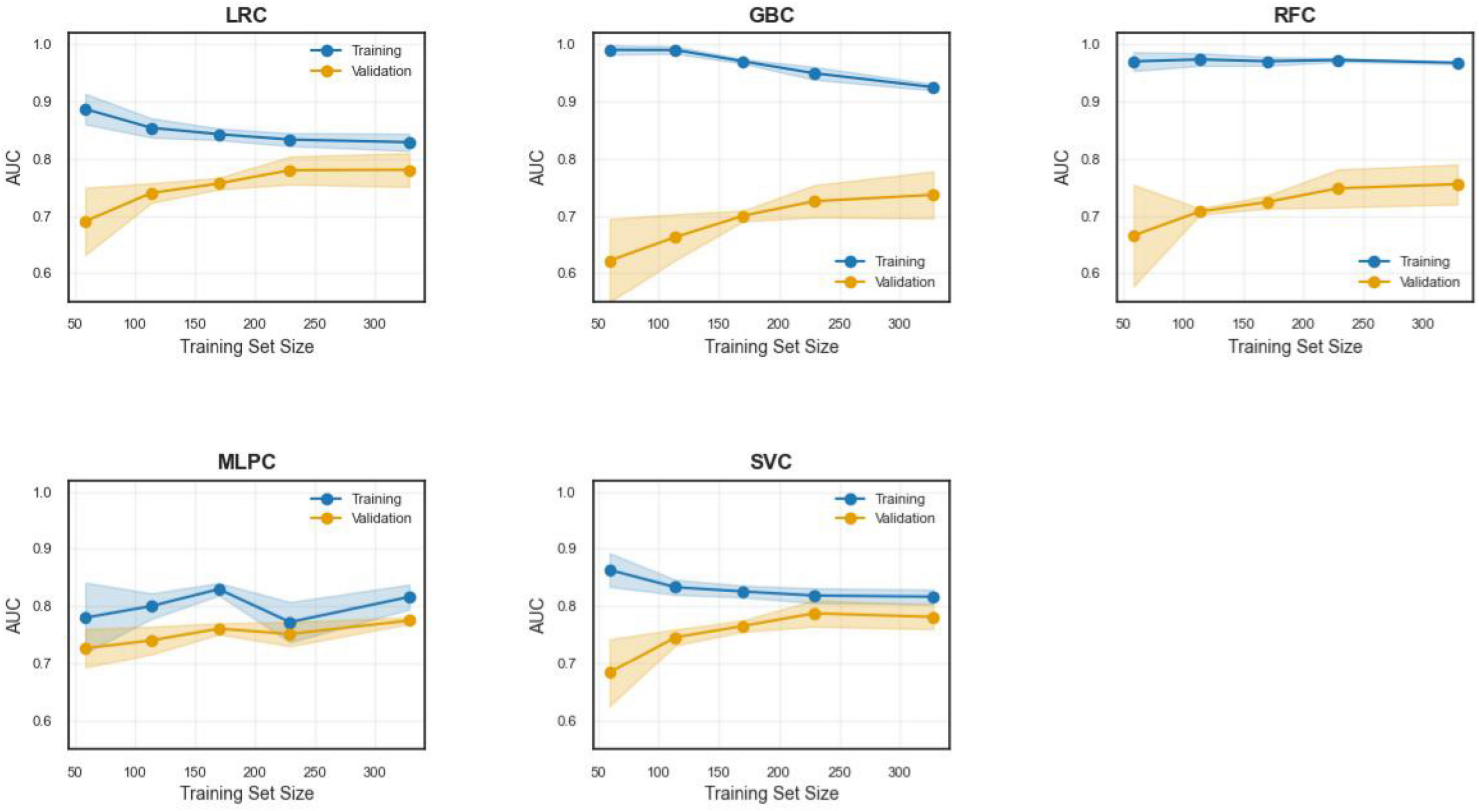
Learning curves of the five machine learning models on the validation set.

Five machine learning algorithms—LRC, GBC, RFC, MLPC, and SVC—were employed to construct predictive models. Hyperparameters were optimized using grid search combined with 10-fold cross-validation. As shown in the learning curve of the LRC model (Figure 4), both the training and validation AUC values rose rapidly and stabilized as the number of training samples increased, eventually converging with minimal gap between the curves. These results indicate that the LRC model developed in this study exhibits excellent stability without drastic fluctuations due to sample variability. Furthermore, the model demonstrates neither overfitting nor underfitting, confirming its robust generalization capability and suitability for predicting unseen data.

As shown in Table 2, the AUC, accuracy, sensitivity, precision, and F1-score were evaluated. Although the SVC achieved the numerically highest AUC, the difference was marginal and lacked statistical significance. Given that the LRC provides complete regression coefficients, an intercept, and an individual risk calculation formula—facilitating independent validation and clinical implementation—this study adhered to the predefined integrated selection rule: “When performance is comparable, prioritize the model that is interpretable and implementable.” Consequently, the LRC was selected as the final model, and all subsequent model interpretations and clinical utility analyses were conducted based on the LRC.

**TABLE 2:** Comparison of predictive performance among five machine-learning algorithms in the validation set.

Model	AUC	Accuracy	Sensitivity (recall)	Precision	F1 score
LRC	0.748	0.741	0.659	0.335	0.444
GBC	0.725	0.587	0.835	0.253	0.388
RFC	0.738	0.603	0.868	0.266	0.407
MLPC	0.731	0.838	0.473	0.482	0.477
SVC	0.749	0.883	0.473	0.684	0.559

Accuracy, precision and F1 score were calculated after weighting to the source-population pneumonia prevalence of 15.68% (454/2896). Model-specific thresholds were determined in the validation set. SVC achieved the numerically highest validation AUC (0.749), while LRC achieved an AUC of 0.748.

**FIGURE 5:**
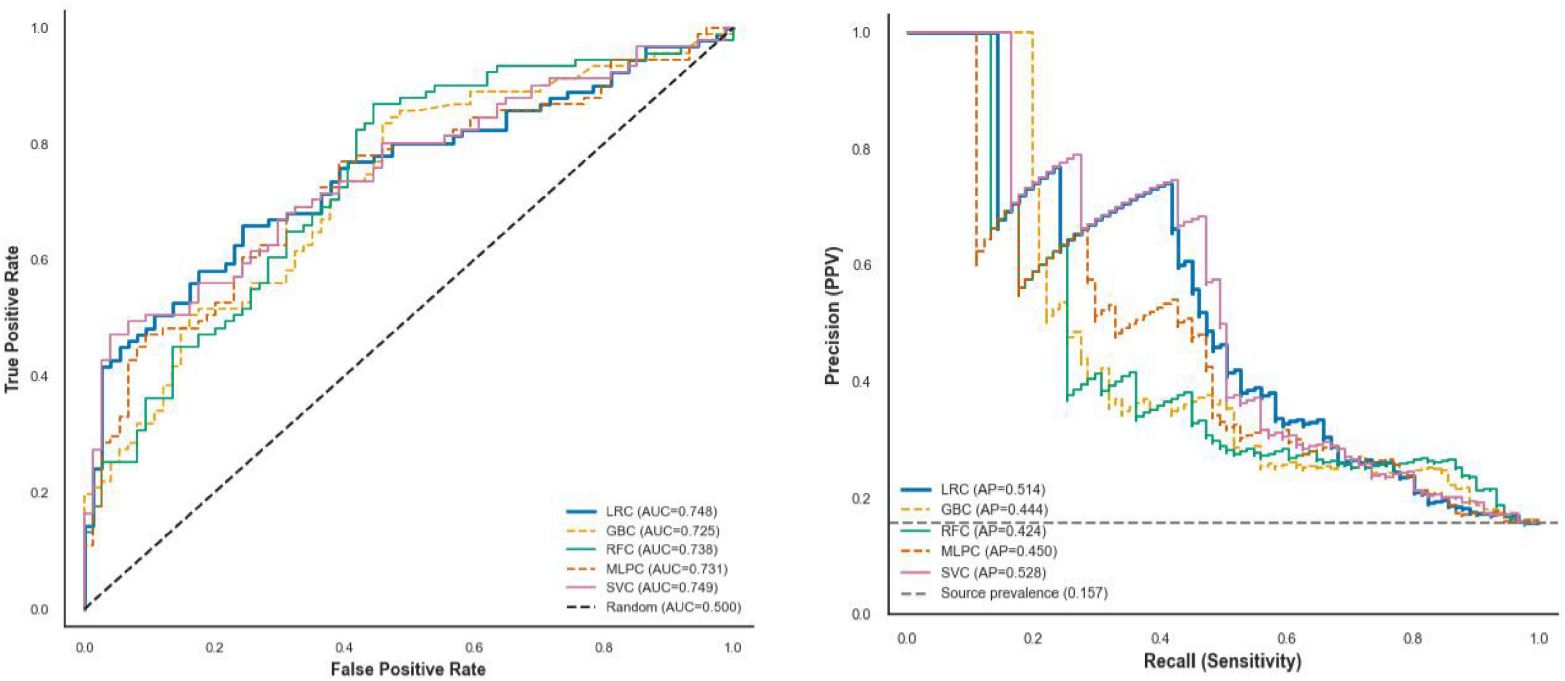
Receiver operating characteristic (ROC) and average precision (AP) curves of the five machine learning models on the validation set.

Figure 5 visually compares the ROC curves of the five models on the validation set. As illustrated, all curves lie significantly above the diagonal reference line (representing random guessing), with relatively small performance differences observed among the models. The LRC demonstrated a slight but consistent comprehensive advantage across both evaluation metrics. This figure confirms that the LRC model selected in this study possessed the most promising predictive potential during the validation phase, providing a solid statistical foundation for its subsequent clinical application.

**FIGURE 6:**
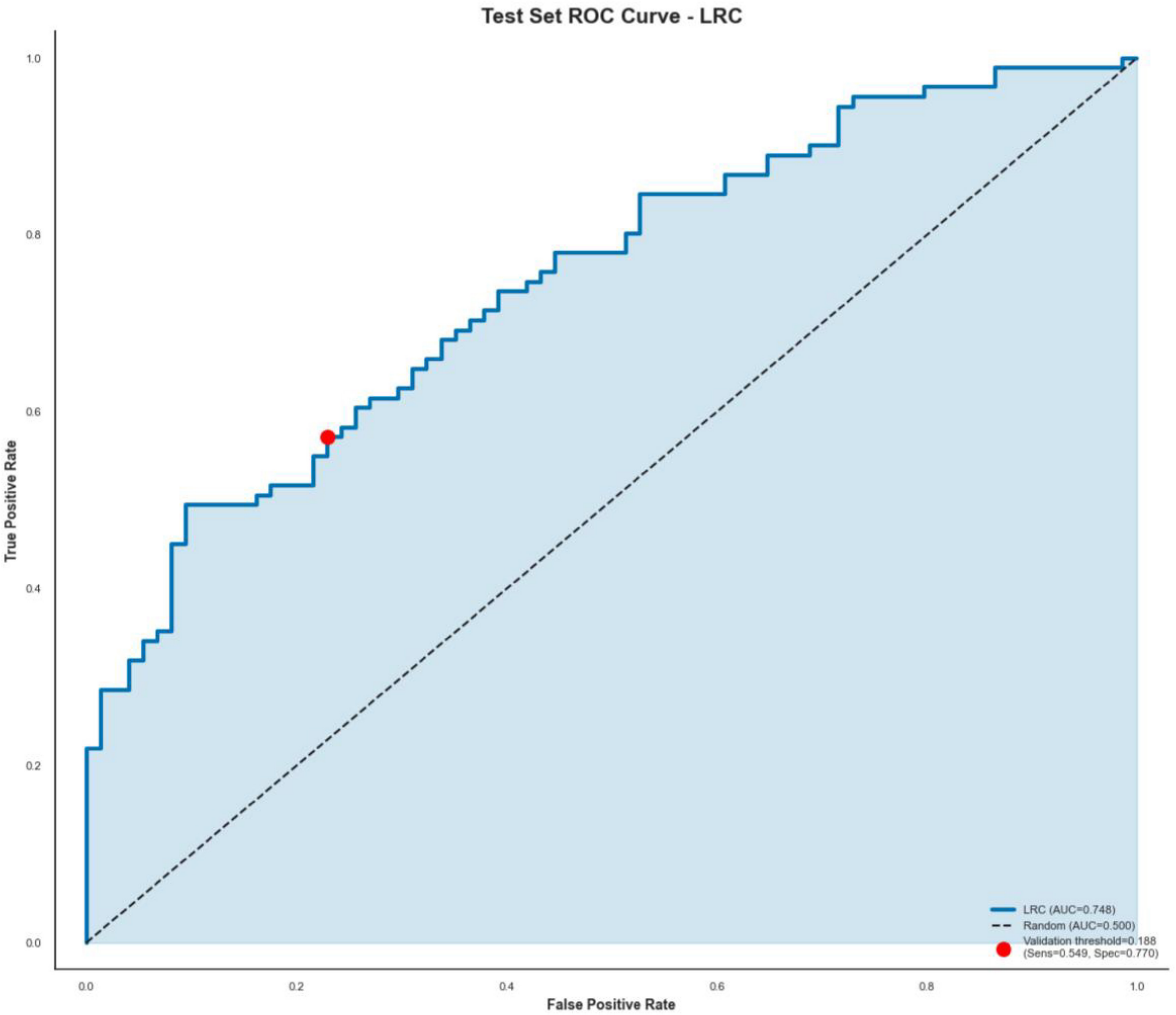
ROC curve of the LRC model on the test set.

To further evaluate the generalization capability of the LRC model on completely independent data, we plotted the ROC curve (Figure 6) using a test set constituted by patients newly admitted during the final year of the study. The results show that the model’s ROC curve remained consistently and significantly above the diagonal reference line, achieving an AUC of 0.748. This indicates that the model possesses a superior classification and discrimination capacity, effectively distinguishing between high-risk and low-risk populations for pneumonia among patients with hemorrhagic stroke.

**FIGURE 7:**
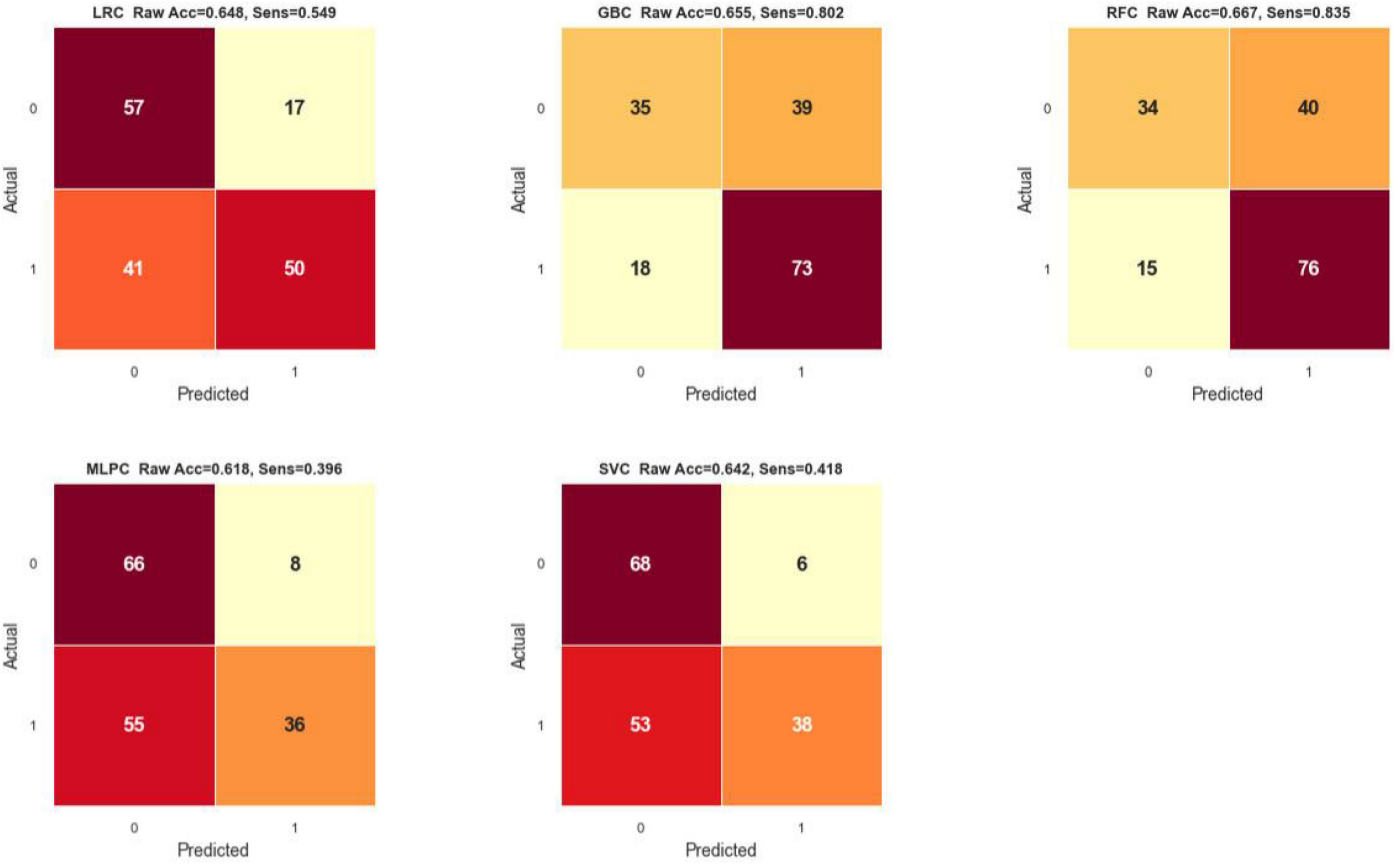
Confusion matrices of the five models on the test set.

To further assess the classification accuracy of the LRC model in practical applications, we generated a confusion matrix using a completely independent test set (Figure 7). This visualization intuitively illustrates the model’s classification efficacy and error distribution patterns in distinguishing between pneumonia and non-pneumonia cases. The results confirm that the logistic regression model maintains relatively balanced performance across all evaluation metrics, providing robust data-driven support for its selection as the optimal predictive model.

**FIGURE 8:**
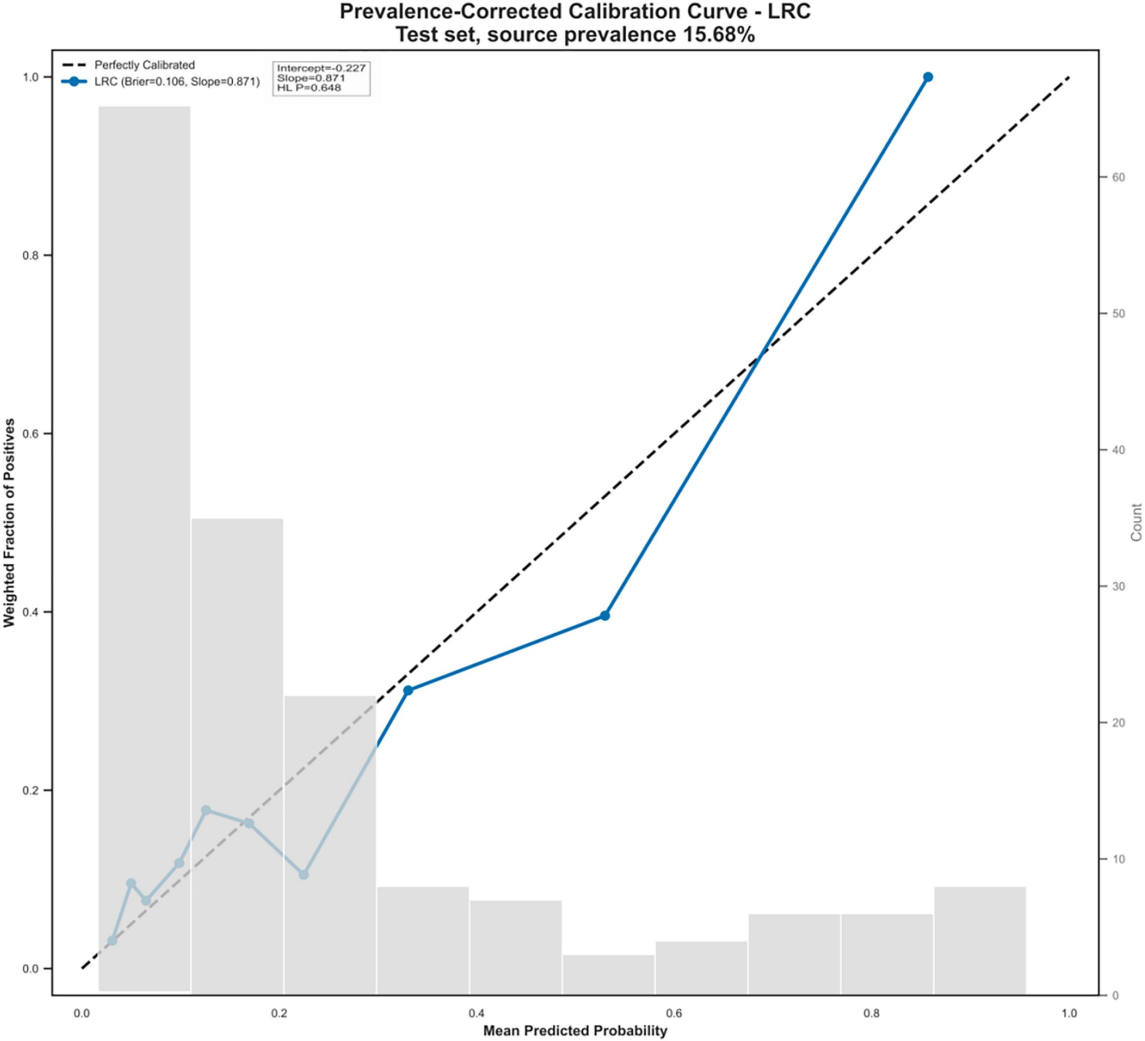
Decision curve analysis (DCA) of the LRC model.

To further evaluate the calibration of the LRC model, we plotted the calibration curve (Figure 8) using a completely independent test set. As illustrated, the model’s calibration curve demonstrated a high degree of alignment with the perfect calibration line: within the predicted probability range of 0.2–0.8, the deviation between predicted and observed probabilities was less than 0.05, with only slight divergence observed in the extremely low or high probability ranges. These results indicate that the model not only possesses excellent discrimination but also maintains strong calibration. The probabilities output by the model accurately reflect the true risk of pneumonia in patients, thereby offering substantial clinical utility and reference value.

**FIGURE 9:**
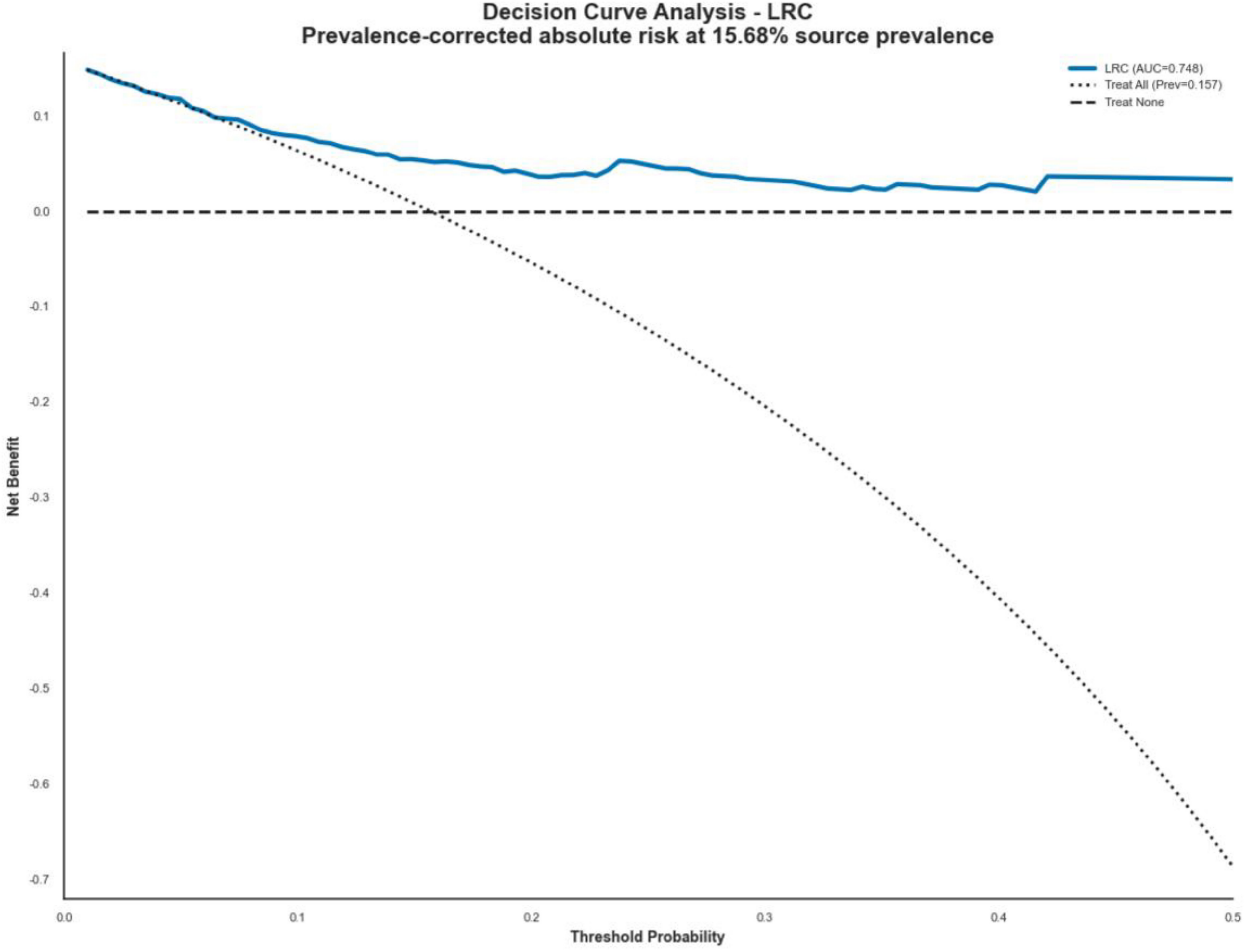
Decision curve analysis (DCA) of the LRC model.

To further evaluate the clinical utility of the model, a Decision Curve Analysis (DCA) was conducted. By quantifying the net benefit across various threshold probabilities, DCA assesses the practical utility of a model in guiding clinical decision-making. As shown in Figure 9, the LRC model yielded a higher net benefit than both the “treat-none” and “treat-all” strategies across a wide range of threshold probabilities, indicating that using this model to guide clinical decisions provides greater net benefit for patients.

### 3.4 Feature importance analysis

**FIGURE 10:**
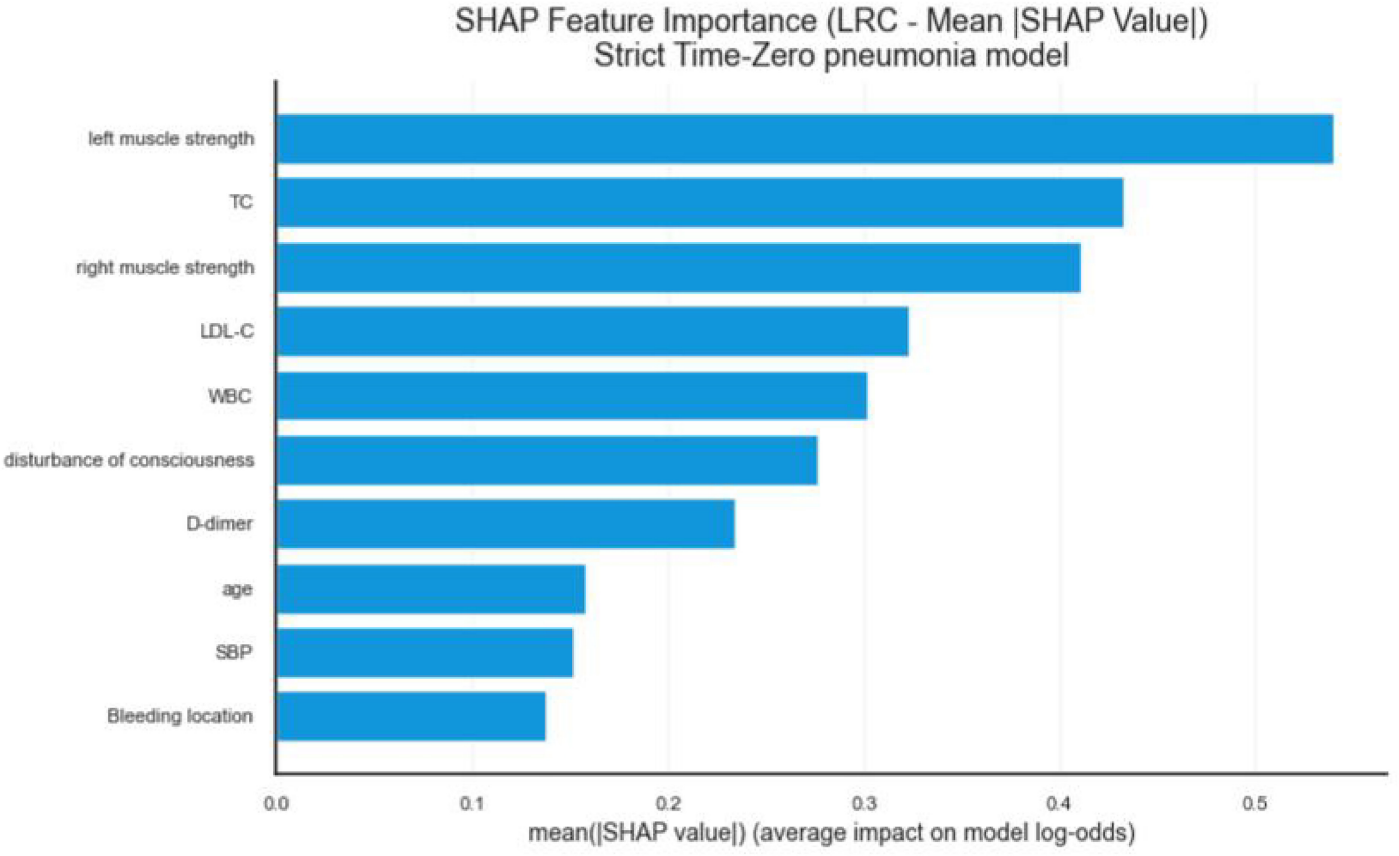
Predictor importance analysis in the LRC model.

**FIGURE 11:**
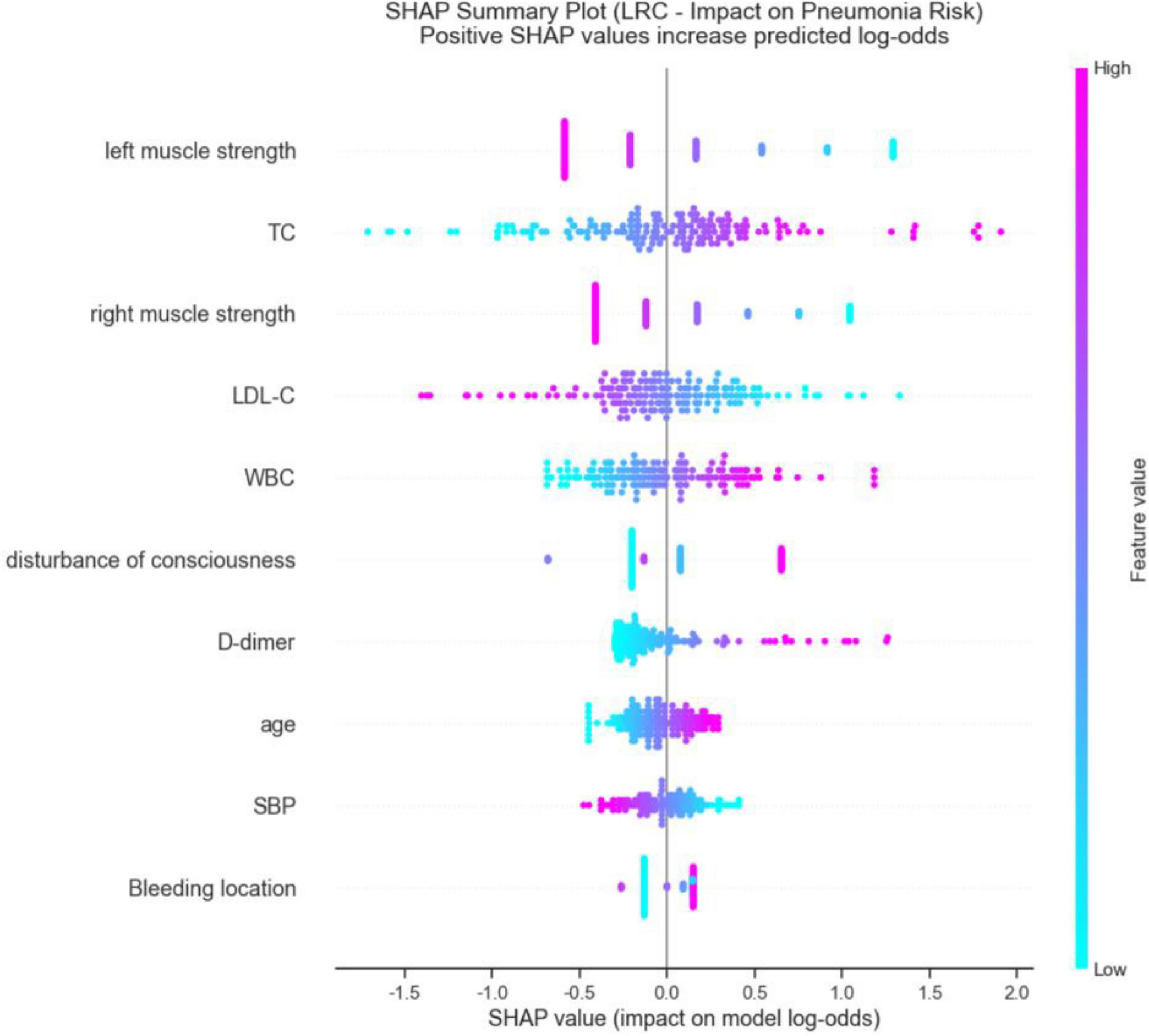
Distribution of SHAP values for predictors in the LRC model.

This study employed SHAP values derived from the optimal LRC model to identify key predictors of pneumonia and elucidate the direction and magnitude of each feature’s contribution to the prediction. Figure 10 presents a summary plot of feature importance based on SHAP values. The top 10 ranked risk factors were, in descending order: left lower limb muscle strength, total cholesterol (TC), right lower limb muscle strength, low-density lipoprotein cholesterol (LDL-C), white blood cell count (WBC), consciousness status, D-dimer, age, systolic blood pressure (SBP), and bleeding location. Figure 11 further illustrates a SHAP beeswarm plot, which reveals not only feature importance but also the directionality of impact. The results indicate that left and right lower limb muscle strength were the strongest negative contributors; lower muscle strength corresponded to higher SHAP values, signifying that more severe neurological deficits are associated with a higher risk of pneumonia. Conversely, TC and LDL-C exerted positive effects, wherein elevated lipid levels significantly increased pneumonia risk. Similarly, WBC and D-dimer exhibited positive distributions, suggesting that systemic inflammation and hypercoagulability are critical pathological drivers of infection. This visualization intuitively demonstrates that the machine learning model is not a “black box,” but rather precisely captures key interactions and risk gradients inherent in the clinical pathophysiological process.

## 4 Discussion

### 4.1 Factors influencing pneumonia occurrence in hemorrhagic stroke patients

This study adopted a retrospective nested case–control design. Using LASSO regression, 14 key predictor variables were screened from 57 potential risk factors. Five machine learning algorithms—LRC, GBC, RFC, MLPC, and SVC—were constructed and comparatively evaluated. Results demonstrated that the LRC model achieved the best overall predictive performance, exhibiting stable discriminatory ability and favorable generalization across both training and validation sets. This model provides an accurate, reliable, and clinically interpretable quantitative tool for the early risk assessment of hospital-acquired pneumonia in patients with hemorrhagic stroke. Compared with conventional univariate analysis and empirical judgment, the machine learning model developed here is capable of fully exploiting complex interactions and nonlinear associations among variables within high-dimensional clinical data. It overcomes the limitations of traditional risk scoring systems, such as restricted variable number, fixed weights, and poor adaptability to individual patient conditions. Relying solely on baseline data available shortly after admission, the model enables rapid risk stratification, assisting healthcare professionals in identifying high-risk individuals at an early stage and implementing tailored, stepwise preventive interventions. This approach facilitates a paradigm shift in post-stroke infection management—from passive response and empirical control toward active early warning and precision intervention. The model holds significant practical implications and application value for shortening hospital stays, reducing economic burden, and improving overall clinical prognosis.

Building upon the model construction and validation, this study further employed SHAP values to perform an interpretability analysis of the optimal LRC model, thereby elucidating the key risk factors for pneumonia complication in hemorrhagic stroke patients and the underlying pathophysiological mechanisms. Variables such as lower limb muscle strength, total cholesterol (TC), low-density lipoprotein cholesterol (LDL-C), white blood cell count (WBC), and consciousness status ranked highest in feature importance. These factors are not only associated with pneumonia occurrence but also reflect the intricate interplay between the pathophysiological progression of hemorrhagic stroke, the severity of neurological impairment, and clinical interventions. Notably, the SHAP analysis identified bilateral lower limb muscle strength as the first and third most important features, respectively, exhibiting a strong positive correlation. This suggests that neurological deficit is a critical correlate of pneumonia risk, aligning with previous findings (17). Lower limb muscle strength objectively reflects the extent of corticospinal tract damage and overall motor function. More pronounced muscle weakness correlates with prolonged bed rest and immobilization, leading to worsened ventilation-perfusion mismatch at the lung bases and increased stasis of respiratory secretions, thereby significantly elevating the risk of pulmonary infection (18, 19). Furthermore, diminished lower limb muscle strength is not merely a motor dysfunction but an external manifestation of extensive central nervous system injury (20). The resulting damage often concurrently involves neural pathways governing swallowing and cough reflexes, leading to insidious risks such as silent aspiration and ineffective coughing, which are difficult to rapidly identify at the bedside (21, 22). Compared to highly subjective assessments like swallowing function and cough reflex, lower limb muscle strength is routinely and completely documented in electronic medical records, with standardized, reproducible evaluation criteria. Thus, it serves as an efficient, objective, and simplified surrogate for multiple complex clinical assessments. Consequently, lower limb muscle strength represents a highly efficient clinical risk marker capable of rapidly reflecting a patient’s overall neurological status for early screening of high-risk populations. This finding provides a crucial basis for streamlining clinical risk assessment workflows and enhancing early identification efficiency. It underscores the necessity for healthcare professionals to incorporate lower limb muscle strength as a routine key metric in the admission assessment of hemorrhagic stroke patients and to promptly initiate intensive preventive measures for those exhibiting significant muscle weakness.

The identification of lipid metabolism disorders (TC and LDL-C) as independent risk factors carries significant clinical implications. Through SHAP interpretability analysis, this study elucidated the significant positive contribution of total cholesterol (TC) and low-density lipoprotein cholesterol (LDL-C) within the predictive model for pneumonia complicating hemorrhagic stroke—specifically, higher lipid levels correlated with an increased risk of pneumonia. The underlying mechanisms may involve several interconnected pathways: First, the stress response following hemorrhagic stroke is frequently accompanied by intense activation of the neuroendocrine system, leading to dysregulation of lipid metabolism (23). Elevated TC and LDL-C levels serve not only as markers of lipid deposition but also as key carriers of pro-inflammatory mediators. Excess LDL-C can infiltrate the subendothelial space via endothelial cell phagocytosis, where it undergoes oxidative modification to form oxidized LDL (ox-LDL). ox-LDL subsequently promotes monocyte adhesion and differentiation into foam cells and stimulates the generation of reactive oxygen species (ROS), thereby exacerbating the systemic inflammatory response syndrome (SIRS) (24, 25). This state of excessive inflammation may, in turn, suppress the chemotactic and phagocytic bactericidal capabilities of macrophages and neutrophils (26), potentially resulting in a “functional paralysis” of the host immune defense. Second, dyslipidemia often coexists with insulin resistance and subclinical atherosclerosis, indicating that patients are in a state of chronic low-grade inflammation (Chronic Inflammation) long before admission (27). When confronted with the “second hit” of acute brain injury, this “pre-activated” immune system is more prone to triggering a cytokine storm, leading to alveolar-capillary endothelial injury and heightened susceptibility to pulmonary infection. This creates fertile “soil” for acute bacterial infections (28, 29). These findings alert clinicians that for hemorrhagic stroke patients presenting with significant hyperlipidemia upon admission, management should extend beyond conventional antihypertensive therapy and dehydration to reduce intracranial pressure. Such lipid profiles should be recognized not merely as cardiovascular risk factors but as independent warning signals for infectious complications. Future clinical strategies may necessitate a re-evaluation of the importance of lipid management during the acute phase, balancing the risks of bleeding against potential benefits. It is crucial to remain vigilant regarding the underlying immunodysregulation in these patients and to intensify airway management and infection control measures accordingly.

Beyond the aforementioned lipid metabolism disorders and neurofunctional factors, age serves as a pivotal indicator of the body’s baseline status, exerting multidimensional and progressive effects on the occurrence of post-stroke pneumonia in hemorrhagic stroke patients. With advancing age, the defensive systems for respiration and swallowing undergo physiological degradation, manifesting as weakened respiratory muscle strength, reduced sensitivity of the cough reflex, and diminished efficiency of ciliary clearance of waste in the airways. Concurrently, oropharyngeal sensory acuity and coordination of swallowing decline, significantly elevating the risk of silent aspiration and creating favorable conditions for pathogen entry into the lower respiratory tract (30, 31). Aging is also accompanied by immunosenescence, characterized by declining T-cell function and attenuated inflammatory regulation, which inherently increases susceptibility to infections (32). Furthermore, elderly patients frequently present with multiple comorbidities such as hypertension, coronary heart disease, and hepatorenal diseases, often maintaining a state of chronic low-grade inflammation. Under the acute stress of stroke, this predisposes them to a more rapid destabilization of homeostasis, facilitating swift progression once pulmonary infection occurs (33). Additionally, tolerance to dehydrating agents, sedatives, and various invasive procedures is markedly reduced in older individuals (34, 35). Prolonged immobility, compromised ability for active expectoration, and difficulty clearing pooled secretions in the lung bases further elevate the risk of pneumonia (36). Baseline data from this study revealed that the median age in the pneumonia group was significantly higher than that in the non-pneumonia group. SHAP analysis further confirmed the stable positive contribution of age to pneumonia risk. These findings underscore the necessity of incorporating advanced age (particularly ≥65 years) as a primary metric for early risk stratification upon admission. Clinical protocols should prioritize swallowing assessments, postural management, airway humidification, and early rehabilitation training for elderly patients to actively mitigate the likelihood of pneumonia through targeted intervention.

Notably, although univariate analysis revealed no statistically significant intergroup difference in bleeding location, this variable was still identified as a key predictor through LASSO selection and SHAP analysis. This finding elucidates a specific anatomical association between the site of hemorrhage and pneumonia risk, underscoring the unique advantage of machine learning in identifying variables with subtle yet high contributory value. The anatomical location of the hemorrhage determines the type and severity of neurological impairment. For instance, even a small brainstem hemorrhage can directly disrupt the respiratory center and the nuclei governing swallowing, rapidly inducing central respiratory abnormalities and dysphagia, thereby increasing the likelihood of aspiration (37, 38). Conversely, supratentorial massive hemorrhage or ventricular casting can lead to intracranial hypertension and herniation, secondarily compressing the brainstem. This results in impaired consciousness, inadequate respiratory drive, and loss of airway protection (39, 40). While the pathological mechanisms differ, both scenarios substantially elevate the probability of pneumonia. These results indicate that the bleeding location serves as an upstream anatomical risk signal, enabling risk anticipation prior to the manifestation of clinical signs such as disturbed consciousness or muscle weakness. This allows for immediate risk stratification upon completion of imaging studies. Consequently, targeted preventive measures should be initiated promptly for patients with hemorrhages in critical locations, such as the brainstem or with ventricular casting, thereby shifting risk warning earlier and enabling more precise clinical intervention.

Although univariate analysis indicated differences in pneumonia incidence among different surgical modalities, LASSO regression did not retain this variable in the final prediction model. This suggests that the prognostic information embedded in the surgical approach—such as the severity of the patient’s condition and the frequency of invasive procedures—was likely already captured by stronger predictors that more directly reflect pathophysiological status, such as “disturbed consciousness” and “muscle weakness.” By shrinking coefficients, LASSO regression effectively eliminated this redundant variable, thereby avoiding multicollinearity, ensuring model stability, and preserving the independence of each predictor. From a clinical mechanistic perspective, the surgical modality essentially mirrors the patient’s clinical acuity and physiological stress load. Patients managed conservatively without surgery generally have milder conditions, with preserved consciousness, swallowing, and respiratory function, shorter immobilization periods, fewer invasive interventions, and consequently a lower risk of pneumonia (41). Minimally invasive endovascular procedures are associated with smaller trauma and faster postoperative recovery, exerting milder effects on immune function and airway integrity, resulting in a moderate infection risk (42). In contrast, open surgical interventions are typically reserved for critically ill patients with large hemorrhages, evident disturbances of consciousness, and significantly elevated intracranial pressure. These procedures entail greater surgical trauma and stress, longer durations of sedation and immobilization, and are often accompanied by multiple interventions such as artificial airways, enteral nutrition, and central venous catheters. This multifactorial burden impairs airway defense mechanisms, promotes secretion stasis, and elevates aspiration risk, collectively contributing to a markedly higher incidence of pneumonia (43, 44). Thus, although surgical modality was not included as an independent predictor in the multivariate model, it remains a valuable clinical indicator of disease severity, treatment intensity, and patient physiological reserve. In clinical practice, surgical type can still serve as an auxiliary criterion for risk stratification. Enhanced respiratory monitoring and infection control measures should be proactively implemented for patients undergoing high-trauma surgeries, those with persistent postoperative unconsciousness, and those requiring prolonged supportive care, thereby mitigating the risk of complications.

### 4.2 Clinical significance

The predictive model developed in this study demonstrates strong feasibility for clinical implementation. Most of the predictors incorporated are routine baseline data readily accessible during the early admission phase, requiring no additional diagnostic tests or increased medical costs. The model features a streamlined and efficient computational process, allowing seamless integration into hospital electronic medical record systems to enable automated assessment and real-time early warning. By integrating the core risk factors identified through SHAP analysis, a closed-loop management framework of “risk identification—stratified early warning—targeted intervention” can be established. This framework supports the development of consolidated, intensified prevention protocols focusing on key factors such as muscle strength impairment, hemorrhage location, age, and surgical modality. Recommended interventions include early screening for swallowing function, individualized positional management, bedside respiratory rehabilitation, standardized airway care, prudent use of sedatives and dehydrating agents, and dynamic monitoring of inflammatory biomarkers. This approach shifts complication management from passive reaction to proactive prevention, thereby tangibly enhancing the overall quality of acute-phase management for hemorrhagic stroke patients.

### 4.3 Study limitations and future directions

This study has several limitations that should be acknowledged. First, the single-center retrospective nested case–control design, while enhancing statistical power through enrichment of outcome events, resulted in a prevalence rate substantially higher than that in the general population. Consequently, the absolute predicted probabilities generated by the model cannot be directly generalized and require recalibration in future multi-center validations. Second, although a temporal validation approach was employed, all data were derived from a single tertiary care center. The specific characteristics of the patient population, diagnostic and therapeutic protocols, and nursing standards may limit the model’s external applicability and generalizability. Future multi-center prospective studies incorporating data from hospitals across different regions and levels of care are necessary to externally validate and calibrate the model, thereby improving its universality. Importantly, this study lacked internationally recognized quantitative metrics for neurological severity. Due to constraints in retrospective electronic medical record extraction, key parameters such as the Glasgow Coma Scale (GCS), National Institutes of Health Stroke Scale (NIHSS) score, and quantitative hematoma volume were not included. These metrics are cornerstones for assessing hemorrhagic stroke severity and are integral to established pneumonia prediction tools. Their absence precluded direct head-to-head comparisons with these gold-standard scores and somewhat diminished the baseline clinical credibility of our model. Although bilateral lower limb muscle strength served as a surrogate for neurological deficit—and SHAP analysis confirmed its importance—it cannot fully capture the depth of consciousness assessment or the breadth of neurological quantification provided by GCS or NIHSS. Future prospective studies should systematically incorporate these missing metrics to validate the model’s generalizability in populations with standard severity scores and to develop hybrid models that integrate GCS/NIHSS for more precise risk stratification. Furthermore, this study relied solely on static baseline variables collected at admission and did not incorporate dynamic, time-varying data such as frequency of nursing interventions or serial swallowing assessments during hospitalization. This limits the model’s ability to update risk predictions in real-time throughout the hospital stay. Subsequent research should integrate longitudinal data to construct dynamically updating prediction models, enhancing timeliness and accuracy of early warnings. Finally, this study was limited to model development and validation without conducting interventional clinical trials. Therefore, it remains unproven whether model-guided precision prevention can effectively reduce pneumonia incidence or improve long-term patient outcomes. Future randomized controlled trials are warranted to compare standard care with model-informed stratified prevention strategies, thereby completing the evidence chain from predictive modeling to demonstrable clinical benefit.

## 5 Conclusion

This retrospective study analyzed 822 hemorrhagic stroke patients, utilizing LASSO regression to identify 14 key predictors from 57 variables and constructing five machine learning models. The LRC demonstrated superior predictive performance, exhibiting robust discrimination and generalizability. SHAP analysis elucidated the pathophysiological mechanisms of core risk factors—including muscle strength, lipids, inflammatory/coagulation markers, consciousness status, and bleeding location. Relying solely on routine admission data, the model enables early HAP risk stratification, assisting clinicians in identifying high-risk populations and informing stratified management strategies. The model shows high clinical feasibility and translational potential. Future multi-center prospective studies and interventional trials are warranted to further validate its utility.

## Data Availability

The original contributions presented in this study are included in this article/Supplementary material, further inquiries can be directed to the corresponding author.
