# Supplementary material for: Construction and validation of a machine learning-based model for predicting pneumonia risk in patients with hemorrhagic stroke
Source: Front Med (Lausanne). 2026 Jul 24;13:1869603. doi: 10.3389/fmed.2026.1869603 (PMC13447289; doi:10.3389/fmed.2026.1869603)
Supplement: Supplementary file 1 [file Data_Sheet_1.PDF]

1.Calibration performance of the final model

Calibration performance of the final model

Dataset	Brier	LogLoss	Calibration intercept	Calibration slope	HL chi-square	df	HL P value
Validation	0.106	0.364	-0.228	0.930	4.649	8	0.794
Test	0.106	0.360	-0.227	0.871	5.992	8	0.648

2.Formula for risk calculation

P(pneumonia) = 1 / (1 + exp(-LP))

LP = 0.3053755366
+ 0.0154025097 * age
- 0.0077277555 * SBP
- 0.3758145611 * left side muscle strength
- 0.2911601127 * right muscle strength
+ 0.2159064281 * D-dimer
+ 0.1160545903 * WBC
- 0.0040194415 * HB
- 1.8774789514 * INR
- 0.9801244793 * LDL-C
+ 0.8438452456 * TC
+ 0.2909565298 * I(Stroke type = 1)
+ 0.2777125688 * I(Bleeding location = 1)
+ 0.2234066221 * I(Bleeding location = 2)
+ 0.1303052034 * I(Bleeding location = 3)
- 0.1320214567 * I(Bleeding location = 4)
+ 0.2798037524 * I(Bleeding location = 5)
+ 0.5365819127 * I(liver and kidney disease = 1)
+ 0.2777120114 * I(disturbance of consciousness = 1)
- 0.4808526562 * I(disturbance of consciousness = 2)
+ 0.0692283733 * I(disturbance of consciousness = 3)
+ 0.8545264959 * I(disturbance of consciousness = 4)

Formula note: I(condition)=1 if the condition is true and 0 otherwise. The reference category for Stroke type, Bleeding location and disturbance of consciousness is code 0. Predicted risk >=0.188 was classified as high risk. Continuous variables were median-imputed and clipped to the training-set 1st/99th percentiles before calculation.

3. Examples of Key Thresholds in DCA and Clinical Impact Curves

Threshold	Model net benefit	Treat-all net benefit	High risk per 1000	True positives per 1000	False positives per 1000
0.050	0.118	0.113	765.2	149.9	615.3
0.099	0.078	0.064	521.1	122.3	398.8
0.149	0.054	0.010	348.9	98.2	250.7
0.188	0.041	-0.039	279.9	86.1	193.7
0.198	0.039	-0.052	266.7	84.4	182.3
0.302	0.032	-0.208	130.4	62.0	68.4
0.401	0.027	-0.408	84.1	50.0	34.2
0.500	0.033	-0.686	56.2	44.8	11.4

4.Clinical Impact Curve

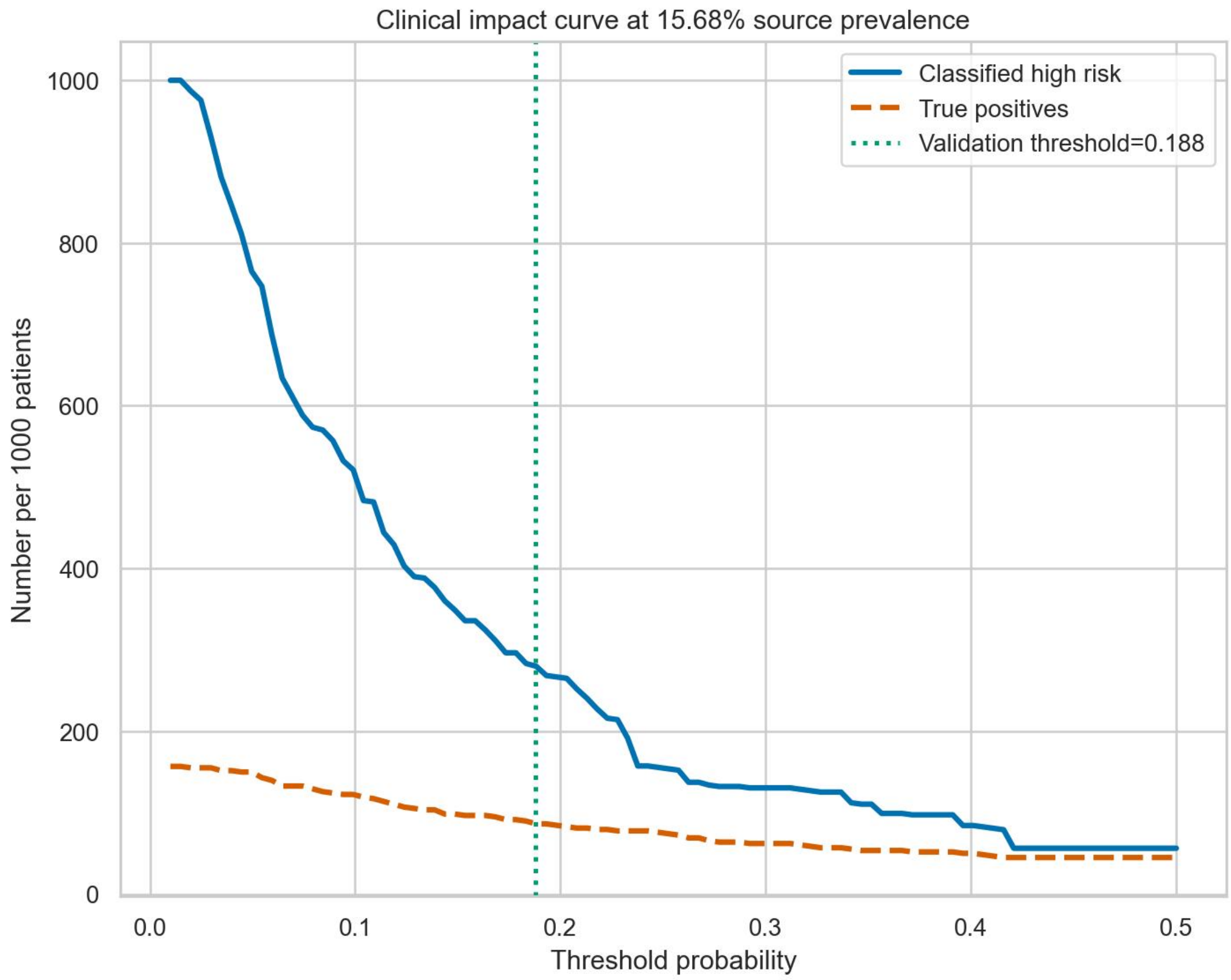

Clinical Impact Curve for the Logistic Regression Model

5.Comparison of Baseline Characteristics Between the Included Patients

Tabal 1 Baseline Characteristics of the Included Patients ［n（%），M（P_{25}，P_{75}）］

Variables	Pneumonia (n=454)	Non-pneumonia (n=368)	t/χ²/Z	*P*
Type of Stroke			0.424	0.515
Intracerebral Hemorrhage	318(70.04)	250(67.93)		
Subarachnoid Hemorrhage	136(29.96)	118(32.07)		
History of Surgery (within past month)	85(18.72)	60(16.30)	0.818	0.366
History of Anticoagulant/Antiplatelet Therapy	11(2.42)	3(0.81)	3.138	0.076
Smoking History	149(32.82)	127(34.51)	0.261	0.610
Alcohol Consumption History	135(29.74)	108(29.35)	0.015	0.904
Diabetes Mellitus	41(9.03)	25(6.79)	1.378	0.240
Hypoproteinemia	152(33.48)	101(27.45)	3.474	0.062
Systolic Blood Pressure (mmHg)	150(134,166)	151(136,168)	-0.806	0.420
Diastolic Blood Pressure (mmHg)	86(78,98)	86(76,98)	-0.753	0.452
Immobilization status	88(19.38)	70(19.02)	0.017	0.896
Mechanical/Physical prophylaxis (within 48h)	181(39.87)	142(38.59)	0.140	0.708
Use of dehydrating agents	352(77.53)	285(77.45)	0.001	0.976
Use of vasoactive drugs	381(83.92)	317(86.14)	0.782	0.376
Platelet count (×10⁹/L)	193(158,233)	194(167,236)	-1.134	0.257
Activated Partial Thromboplastin Time (s)	25.8(24.58,27.23)	25.8(25.2,27)	-0.909	0.363
International normalized ratio	0.9(0.85,0.95)	0.9(0.86,0.94)	-0.903	0.367
Prothrombin time ratio	0.91(0.86,0.96)	0.9(0.87,0.93)	-1.931	0.053
Triglycerides (mmol/L)	1.41(1.25,1.62)	1.42(1.19,1.63)	-0.301	0.764
Total cholesterol (mmol/L)	4.38(3.92,4.71)	4.47(4.04,4.75)	-1.821	0.069

Variables	Pneumonia (n=454)	Non-pneumonia (n=368)	t/χ²/Z	*P*
Serum creatinine (μmol/L)	68(60,83)	68(60,79)	-0.739	0.460

Note:"-" indicates Fisher's exact probability method was used. Continuous variables are presented as mean ± SD or median (IQR); categorical variables are presented as n (%). P < 0.05 indicates statistical significance. SBP: Systolic Blood Pressure; DBP: Diastolic Blood Pressure.

5.Comparison of Predictive Performance Metrics Among Five Machine Learning Algorithms

Tabal 2 Comparison of Predictive Performance Metrics Among Five Machine Learning Algorithms

Model	Dataset	AUC (95% CI)	Sensitivity (95% CI)	Specificity (95% CI)	Accuracy	Precision	NPV	F1	Brier	Reference	AUC difference vs LRC	P value vs LRC
LRC	Validation	0.748 (0.677-0.821)	0.659 (0.558-0.755)	0.757 (0.656-0.852)	0.741	0.335	0.923	0.444	0.106	LRC		Reference
GBC	Validation	0.725 (0.641-0.800)	0.835 (0.756-0.907)	0.541 (0.429-0.657)	0.587	0.253	0.946	0.388	0.115	LRC	-0.024	0.298
RFC	Validation	0.738 (0.658-0.812)	0.868 (0.798-0.933)	0.554 (0.435-0.667)	0.603	0.266	0.958	0.407	0.116	LRC	-0.01	0.642
MLPC	Validation	0.731 (0.652-0.806)	0.473 (0.369-0.578)	0.905 (0.831-0.970)	0.838	0.482	0.902	0.477	0.118	LRC	-0.017	0.148
SVC	Validation	0.749 (0.675-0.819)	0.473 (0.366-0.580)	0.959 (0.909-1.000)	0.883	0.684	0.907	0.559	0.105	LRC	0.001	0.889
LRC	Test	0.748 (0.674-0.816)	0.549 (0.449-0.656)	0.770 (0.671-0.862)	0.736	0.308	0.902	0.395	0.106	LRC		Reference
GBC	Test	0.739 (0.661-0.811)	0.802 (0.713-0.882)	0.473 (0.361-0.587)	0.525	0.221	0.928	0.346	0.113	LRC	-0.009	0.666
RFC	Test	0.750 (0.675-0.819)	0.835 (0.755-0.906)	0.446 (0.333-0.556)	0.507	0.219	0.936	0.347	0.112	LRC	0.002	0.926
MLPC	Test	0.728 (0.652-0	0.396 (0.294-0	0.892 (0.815-0.	0.814	0.405	0.888	0.4	0.112	LRC	-0.019	0.106

		.804)	.500)	960)								
SVC	Test	0.757 (0.679-0.825)	0.418 (0.320-0.522)	0.919 (0.847-0.974)	0.84	0.489	0.895	0.451	0.105	LRC	0.009	0.260

Note: Predicted probabilities were recalibrated to the source-population prevalence of 15.68%. Accuracy, precision, NPV, F1 and Brier score were prevalence-weighted. AUC differences are calculated as comparator AUC minus LRC AUC. P values are paired bootstrap tests versus LRC.
